# Correction: Hsa-mir-3163 and CCNB1 may be potential biomarkers and therapeutic targets for androgen receptor positive triple-negative breast cancer

**DOI:** 10.1371/journal.pone.0352946

**Published:** 2026-07-06

**Authors:** The PLOS One Editors

There are errors in the caption for Fig 7. Please see the complete, correct Fig 7 caption here.

Fig 7 reports material from the Human Protein Atlas (proteinatlas.org), published under a Creative Commons Attribution-ShareAlike 4.0 International license [2]. No changes were made to the reused content. The images in Fig 7 are not offered under a CC BY license and are therefore excluded from this article’s [1] license.

**Fig 7:**
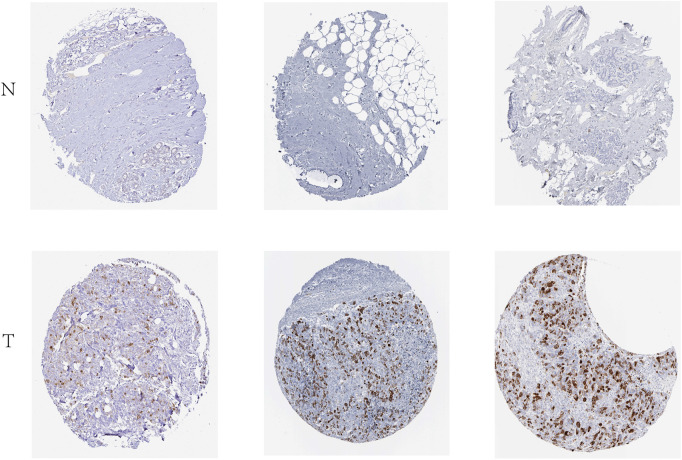
THPA website analysis of CCNB1 protein in BC. CCNB1 protein expression in BC specimens and non-cancerous breast tissues via THPA website analysis. Three representative images of BC (images available from https://www.proteinatlas.org/ENSG00000134057-CCNB1/pathology/breast+cancer#img) and non-cancerous breast tissues were presented (images available from https://www.proteinatlas.org/ENSG00000134057-CCNB1/tissue/breast#img). Image Credit: Human Protein Atlas. N, normal; T, tumor; BC, breast cancer.

## References

[1] Qiu P, Guo Q, Yao Q, Chen J, Lin J. Hsa-mir-3163 and CCNB1 may be potential biomarkers and therapeutic targets for androgen receptor positive triple-negative breast cancer. PLoS One. 2021;16(11):e0254283. doi: 10.1371/journal.pone.0254283 34797837 PMC8604295

[2] Creative Commons. CC BY-SA 4.0 Attribution-ShareAlike 4.0 International [Internet] Available from: https://creativecommons.org/licenses/by-sa/4.0/ (Accessed June 2026)

