# Supplementary figures and images for: Hsa-mir-3163 and CCNB1 may be potential biomarkers and therapeutic targets for androgen receptor positive triple-negative breast cancer
Source: PLoS One. 2021 Nov 19;16(11):e0254283. doi: 10.1371/journal.pone.0254283 (PMC8604295; doi:10.1371/journal.pone.0254283)

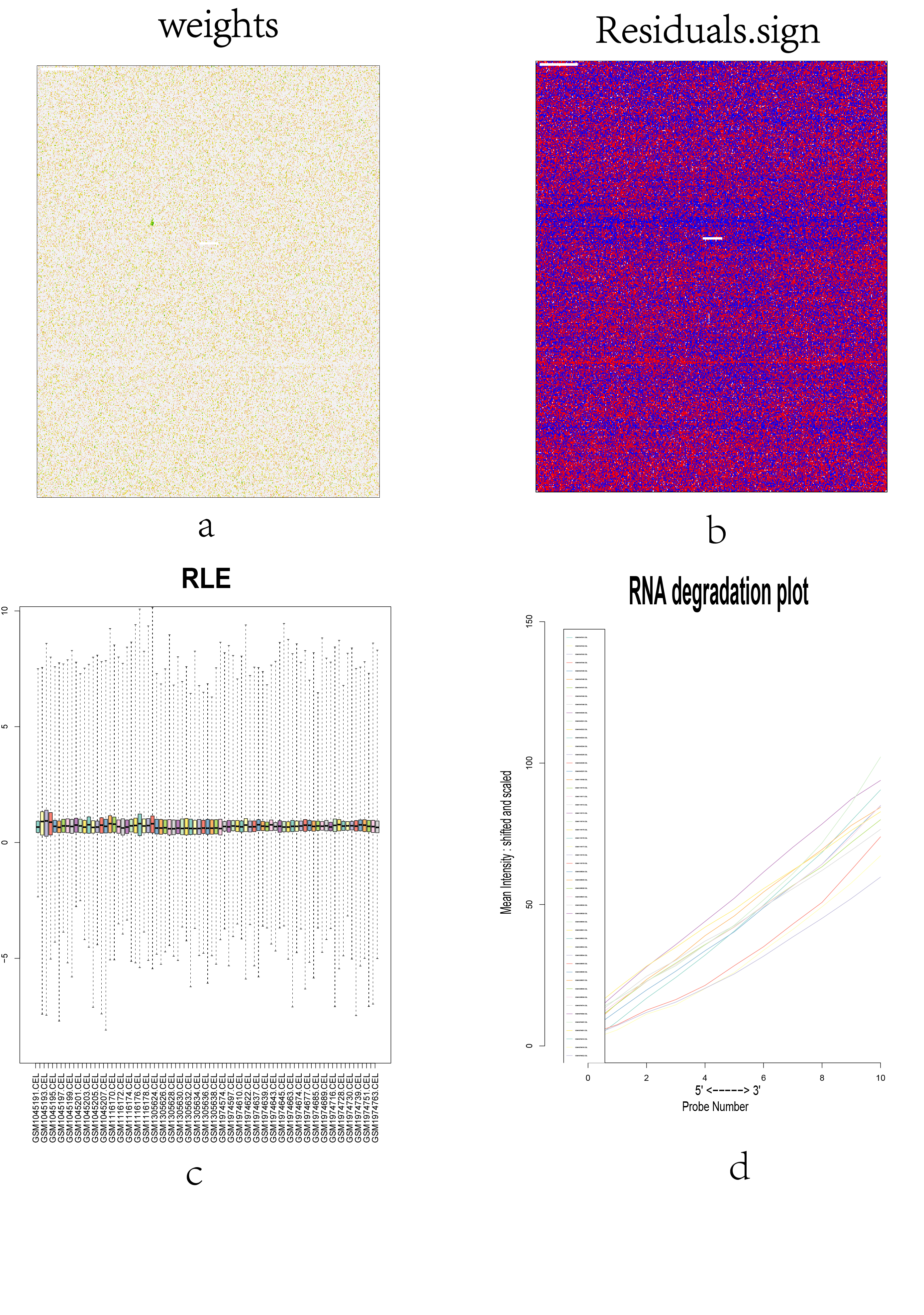

Supplement: S1 Fig — (a) weight map, (b) residual symbol map, (c) relative logarithmic expression map, (d) RNA degradation map. (TIF) [file pone.0254283.s001.tif]
