# Supplementary material for: Hsa-mir-3163 and CCNB1 may be potential biomarkers and therapeutic targets for androgen receptor positive triple-negative breast cancer
Source: PLoS One. 2021 Nov 19;16(11):e0254283. doi: 10.1371/journal.pone.0254283 (PMC8604295; doi:10.1371/journal.pone.0254283)
Supplement: Attachment — Submitted filename: response to reviewers.docx [file pone.0254283.s002.docx]

Dear editor Suhwan Chang,

Firstly, thank you very much for the valuable comments you and the reviewers have made on our manuscript. Unfortunately, we are currently not in a position to carry out the validation trials that the reviewer 1 have suggested. I deeply apologized for our limited conditions. We have modified our manuscript according to the suggestions from reviewers and made it meet PLOS ONE's style requirements. Also, the title has been changed to be more proper. The manuscript with track changes has been re-submitted. Thank you again for your help. We hope that the revised manuscript is approved for publication in PLOS One.

Best regards

Sincerely,

Jianqing Lin

Response to reviewer 1

Thank you for your precious comments and advice. Those comments are all valuable and very helpful for revising and improving our paper, as well as the important guiding significance to our research.

1. As for the Ref.22 from Fujian Medical University, Professor Hong Zhipeng and colleagues identified 7 cell cycle-related genes with unfavorable prognosis in breast cancer. The tumor samples and normal samples from GSE42568, GSE45827 and GSE54002 were all analyzed in this article, and 124 DEGs were identified between the breast tumor samples and normal samples. However, we focused on the AR positive triple-negative breast cancer (TNBC), which is a special subtype of breast cancer. Hence, the DEGs and Hub genes were different between these two studies. TNBC is one of the worst prognosis subtypes of breast cancer due to a lack of effective chemotherapy drugs and targeted drugs. Therefore, the study of TNBC is as important as the study in breast cancer. Furthermore, TNBC has been subdivided into four categories: luminal androgen receptor (LAR), mesenchymal (MES), basal-like immunosuppressed (BLIS), and basal-like immune activated (BLIA). Currently, there are seldom studies in the DEGs, tumor progression and treatment of AR positive TNBC. Besides, the tumor samples included in our study were different from these in Professor Hong’s study. In our study, 37 AR positive TNBC samples from GSE76124 and 17, 11 and 16 normal samples from GSE42568, GSE45827 and GSE54002 were included. The normal samples from these two studies were the same, but tumor samples were different. Based on these two reasons, we consider that the different findings from Professor Hong’s team and our team are reasonable and meaningful. The specific samples (GSMseries) were uploaded as additional materials (文件名). Unfortunately, we are currently not in a position to carry out the validation trials about the 13 Hub genes. I am sorry about the poor condition. We hope to have chances to conduct a wet experiment to further validate the results in the future and share them with all of you. Thanks again for your careful review and precious comments.

2. Thank you for your precious comments and advice. The specificity of microRNA-3163 and CCNB1 towards the implication of being a potential marker for AR positive TNBC were reconsidered and discussed. Also, the references have been modified to be more reasonable. The manuscript with tracked changes have been re-submitted.

3. Thank you for your careful review and precious and advice. We are very sorry for the mistakes in this manuscript and inconvenience they caused in your reading. We have revised our manuscripts according to your advice. The exact series GSEseries of GEO datasets that were used in our study has been list out and uploaded as supplementary file. Figure 1 has been removed from the manuscript and submitted as supplementary material. The Figure 5 as well as the figure legend of it has been modified.

Thank you for reviewing our manuscript again and making valuable suggestions. We hope that the revised manuscript is approved for publication.

Response to reviewer 2

Thank you for your summary. We really appreciate your efforts in reviewing our manuscript. We have studied comments carefully and have made correction which we hope to meet with approval. We have modified the conclusion to be more preciseness. The results of the study were revisited carefully. We are very sorry for the mistakes in this manuscript and inconvenience they caused in your reading. We have revised our manuscripts and modified the typing and grammar errors. Thanks again for your effort. We have re-submitted the new manuscript and supplementary file. We hope that the revised manuscript is approved for publication in PLOS ONE.
